# Revealing the Role of lncRNA CCDC144NL-AS1 and LINC01614 in Gastric Cancer *via* Integrative Bioinformatics Analysis and Experimental Validation

**DOI:** 10.3389/fonc.2021.769563

**Published:** 2022-01-10

**Authors:** Weiwei Sheng, Weihong Zhou, Yundi Cao, Yuejiao Zhong

**Affiliations:** ^1^ Physical Examination Center, Nanjing Drum Tower Hospital, The Affiliated Hospital of Nanjing University Medical School, Nanjing, China; ^2^ Department of Oncology, Affiliated Taikang Xianlin Drum Tower Hospital, Medical School of Nanjing University, Nanjing, China; ^3^ Department of Medical Oncology, Jiangsu Cancer Hospital and Jiangsu Institute of Cancer Research and The Affiliated Cancer Hospital of Nanjing Medical University, Nanjing, China

**Keywords:** lncRNAs, WGCNA, gastric cancer, CCDC144NL-AS1, LINC01614, prognosis

## Abstract

Long non-coding RNAs (lncRNAs) are key regulators in the pathophysiology of gastric cancer, and lncRNAs have been regarded as potential biomarkers and therapeutic targets for gastric cancer. The present study performed the WGCNA analysis of the GSE70880 dataset and aimed to identify novel lncRNAs associated with gastric cancer progression. Based on the WGCNA, the lncRNAs and mRNA co-expression network were constructed. A total of four modules were identified and the eigengenes in different modules were involved in various key signaling pathways. Furthermore, the co-expression networks were constructed between the lncRNAs and mRNA; this leads to the identification of 6 modules, which participated in various cellular pathways. The survival analysis showed that high expression of CCDC144NL antisense RNA 1 (CCDC144NL-AS1) and LINC01614 was positively correlated with the poor prognosis of patients with gastric cancer. The *in vitro* validation results showed that CCDC144NL-AS1 and LINC01614 were both up-regulated in the gastric cancer cells. Silence of CCDC144NL-AS1 and LINC01614 both significantly suppressed the cell proliferation and migration of gastric cancer cells, and also promoted the chemosensitivity of gastric cancer cells to 5-fluorouracil. Collectively, our results suggested that the newly identified two lncRNAs (CCDC144NL-AS1 and LINC01614) may act as oncogenes in gastric cancer.

## Introduction

Gastric cancer represents one of the most common human malignancies, and the occurrence of gastric cancer is region dependent with about 60% of the cases are found in developing regions (1, 2). With the improved living condition and early screening, the new cases of gastric cancer in these regions are decreasing. However, the prognosis of gastric cancer has not been largely improved. For the treatment of gastric cancer surgical resection is the key treatment for gastric cancer, while the five-year overall survival of gastric cancer patients after surgical treatment depends on the tumor stages with ~90% in early-stage and ~20% in advanced stage gastric cancer patients (3). In these patients diagnosed at an advanced stage, chemotherapy instead of surgical resection is considered for alleviating the symptoms in patients, however, chemotherapy only exhibits a modest beneficial effect on patients with metastatic patients, and chemotherapy has been largely limited chemoresistance (4, 5). In this regard, further exploration into the mechanisms underlying gastric cancer progression may be helpful for us to develop novel strategies, to improve the clinical outcomes of gastric cancer patients.

Long non-coding RNAs (lncRNAs) belong to a type of RNA without protein-coding capacity, and lncRNAs are longer than 200 nucleotides in length (6). In the past decade, lncRNA has been extensively examined in various diseases including cancers, due to its diverse biological functions such as regulating cell proliferation, apoptosis, invasion, modulating immunity, and so on (7). In gastric cancer, lncRNAs such as HOX transcript antisense RNA, plasmacytoma variant translocation 1, nuclear enriched abundant transcript 1, maternally expressed 3 and colon cancer associated transcript 1 have been reported to involve in gastric cancer progression and the prognosis of patients with gastric cancer (8–12). Due to the large number of existing lncRNAs in the human genome, more efforts are needed to discover more lncRNAs in regulating the pathophysiology of gastric cancer. Recently, high-throughput screening technology such as RNA-sequencing has been performed to identify novel lncRNAs. In addition, the analysis of high-throughput datasets using different bioinformatic strategies has enabled us to identify lncRNAs more efficiently. For example, Zhao et al., performed the analysis in the Gene Expression Omnibus (GEO) datasets including GSE58828 and GSE27342 and found that LINC00355, a novel lncRNA, induced gastric cancer progression *via* enhancing ubiquitination of p53 (13). Ren et al., performed the weighted gene co-expression network analysis (WGCNA), and the analysis revealed ILF3-AS1 acted as a ceRNA to regulate polypyrimidine tract binding protein 1 by repressing miR-29a expression in gastric cancer (14). Foroughi et al., performed the comprehensive bioinformatic analysis and revealed the tissue-specific down-regulation of prostate cancer associated transcript 18 and LINC01133 in gastric cancer development (15). The role of lncRNAs in the drug resistance of gastric cancer cells has also been demonstrated in various studies. For examples, He et al., showed that mesenchymal stem cells- regulated lncRNA MACC1-AS1enhanced chemoresistance through fatty acid oxidation in gastric cancer (16). Zhang et al., found that lncRNA colorectal neoplasia differentially expressed attenuates chemoresistance in gastric cancer *via* serine and arginine rich splicing factor 6 -regulated alternative splicing of phosphatidylinositol binding clathrin assembly protein (17).

Given the promising role of lncRNAs in gastric development, the present study was undertaken to discover novel lncRNAs correlated with the progression of gastric cancer. In this study, we performed the WGCNA analysis using the GEO dataset GSE70880 and constructed the lncRNAs co-expression network to identify the hub lncRNAs. In this dataset, the expression profiles of cancer and adjacent normal tissues form 76 patients (20 with gastric cancer, 20 with colon cancer, 16 with liver cancer and 20 with lung cancer) were studied by microarray and a set of lncRNAs as well as mRNAs were identified as potential biomarkers general to different types of cancer (18–20). In addition, the identified novel hub lncRNAs were subjected to *in vitro* validation studies. The present study will further advance our understanding of the role of the pathophysiology of gastric cancer.

## Materials and Methods

### Selection of Expression Datasets From GEO Database

The GSE70880 dataset was obtained from the GEO database (19). The dataset contained 20 gastric cancer tissue samples and 20 adjacent normal gastric tissue samples. The quality control of the raw data was analyzed using an array Quality package and the expression datasets were further analyzed using the limma package in R software. For criteria for significantly differentially expressed genes were set at false discovery rate (FDR) < 0.05 and fold changes (FCs) > 2.

### WGCNA

The WGCNA package in the R software was utilized for the analysis of lncRNA mRNA co-expression modules (14, 21, 22). The WGCNA parameters of soft threshold power of the adjacency matrix and the criteria of correlation coefficient square of eigengenes were defined according to the approximate scale-free topology preconditions and the criteria of cut-off of ≥30 genes and cut height = 0.15. The adjacency matrix dissimilarity was 0.2. Then, the WGCNA modules (co-expression network) of eigengenes were identified and the networks correlated with agronomic traits were identified with the criterion of stability correlation p ≤ 0.05.

### Functional Enrichment Analysis

The differentially expressed genes from the analyzed dataset were separately subjected to the enrichment analysis for Gene Ontology (GO; http://www.Geneontology.org/) and KEGG (Kyoto Encyclopedia of Genes and Genomes) pathways. Significant GO and KEGG pathways were identified with the criterion of p < 0.05.

### Survival Analysis of the Patients With Gastric Cancer

For the effects of the lncRNAs on the survival of gastric cancer patients, the Kaplan−Meier analysis was performed by using The Cancer Genome Atlas database. In the database, a total of 352 patients with gastric cancer were included. P<0.05 was considered statistically significant.

### Cell Culture

The gastric cancer cells including AGS and SGC7901 and the human normal gastric epithelial cells (GES-1) were obtained from the Chinese Academy of Sciences (Shanghai, China). The cells were cultured in the RPMI-1640 medium (Sigma-Aldrich, St. Louis, USA) supplemented with 100 mg/ml streptomycin, 10% fetal bovine serum and 100 U/ml penicillin (Sigma-Aldrich). The cells were maintained in a humidified incubator with 5% CO_2_ at 37°C. For the establishment of 5-fluorouracil (5-FU)-resistant SGC7901 cells, the SGC7901/5-FU cell line was generated by using a habitual stepwise method, according to the previous studies (23).

### Design and Synthesis of siRNAs, Cell Transfections With siRNAs

The respective siRNAs for CCDC144NL antisense RNA 1 (CCDC144NL-AS1) and LINC01614 were designed and synthesized by RiboBio (Guangzhou, China), and respective scrambled siRNAs were served as negative controls (NCs). For the cell transfections, the cells were seeded at 1 x 10^6^ cells/well. After cells reached ~80% confluence, cells were transfected with 30 nM respective siRNAs using Lipofectamine Plus Reagent (Invitrogen, Carlsbad, USA) as per the manufacturer’s protocol.

### Quantitative Real-Time PCR (qRT-PCR)

The RNA was extracted from cells by using the Trizol reagent (Sigma-Aldrich) as per the manufacturer’s protocol. The PrimeScript RT reagent Kit (Takara, Dalian, China) was used to reversely transcribe mRNA into cDNA. The real-time PCR was performed on an ABI7500 Real-Time PCR System (Applied Biosystems, Waltham, USA) by using the SYRB Premxi DimerEraser Kit (Takara). The relative expression of the lncRNAs in the cells was normalized to GAPDH and was calculated by using the comparative Ct method.

### Cell Counting Kit-8 (CCK-8) Assay

The CCK-8 assay was determined by using the CCK-8 assay kit (Beyotime, Beijing, China) as per the manufacturer’s protocol. After 0, 24, 48, 72 h siRNA transfections, the proliferation of gastric cancer cells were incubated with CCK-8 reagent, and the cell proliferative index was determined by measuring the absorbance at a wavelength of 450 nm.

### Wound Healing Assay

Cell migration ability was measured using the wound healing assay. Gastric cancer cells were plated into 6-well plates at a density of 1×10^6^ cells/well and cultured until 90% confluence. A micropipette tip was then used to make a perpendicular scratch in the middle of each well. After incubation for 24 h, images were taken and the wound-healing rate was assessed by ImageJ.

### Determination of Chemosensitivity of SGC7901/5-FU Cells to 5-FU

For the determination of chemosensitivity, cells were seeded at 1 x10^5^ cells/well. After that, the cells were incubated with varying concentrations of 5-FU. At 48 h after 5-FU treatment, the cell proliferative index was determined by CCK-8 assay as per the manufacturer’s protocol.

### Statistical Analysis

For the *in vitro* experiments, all the experiments were performed in triplicate. The results were shown as mean ± standard error of the mean. GraphPad Prism software was used to perform the statistical analysis. The differences between different treatment groups were determined by unpaired t-test or one-way analysis of variance followed by Bonferroni’s post-hoc test. P<0.05 was considered statistically significant.

## Results

### Volcano Plot of Differentially Expressed mRNA and lncRNA in Gastric Cancer

**Figure 1:**
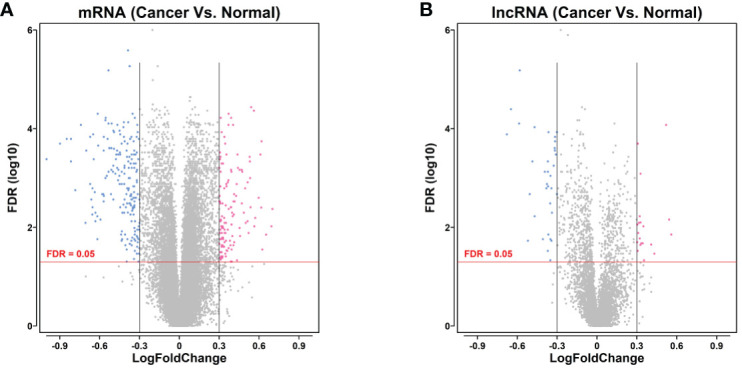
Volcano plot of differentially expressed mRNA and lncRNA in gastric cancer. **(A)** Volcano plot of differentially expressed mRNA of the gastric cancer tissues between gastric cancer group and non-cancerous group. **(B)** Volcano plot of differentially expressed lncRNA of the gastric cancer tissues between gastric cancer group and non-cancerous group. mRNAs or lncRNAs with log_2_FC >2 and FDR <0.05 were shown in red dots; mRNAs or lncRNAs with log_2_FC <−2 and FDR <0.05 were in blue dots. Grey dots represent the non-differentially expressed genes.

Based on the analysis, we performed the bioinformatics analysis, and identified a series of differentially expressed mRNAs (129 upregulated and 187 downregulated) and differentially expressed lncRNAs (19 upregulated and 52 downregulated) between gastric cancer and adjacent normal gastric tissues (**Figures 1A, B**).

### GSEA GO and GSEA KEGG Pathway Analysis of Differentially Expressed mRNA and lncRNA

**Figure 2:**
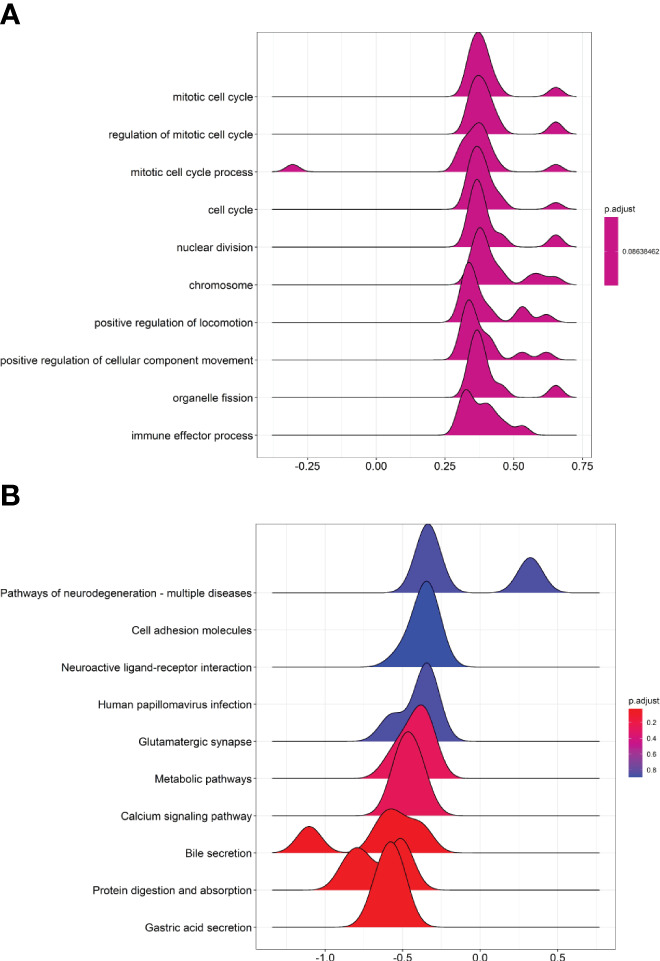
GSEA GO and GSEA KEGG pathway analysis of differentially expressed mRNA and lncRNA. **(A)** Joyplot of GSEA GO analysis. **(B)** Joyplot of GSEA KEGG pathway analysis.

The differentially expressed mRNAs and lncRNAs were further subjected to GSEA GO and KEGG analysis. In the GSEA GO analysis, the mRNA and lncRNAs were mainly enriched in “mitotic cell cycle”, “regulation of mitotic cell cycle”, “mitotic cell cycle process”, “cell cycle”, “nuclear division”, “chromosome” and so on (**Figure 2A**). In the GSEA KEGG analysis, the mRNAs and lncRNAs were mainly enriched in “Pathways of neurodegeneration-multiple diseases”, “cell adhesion molecules”, “neuroactive ligand-receptor interaction”, “human papillomavirus infection”, “glutamatergic synapse” and so on (**Figure 2B**).

### WGCNA of GSE70880

**Figure 3:**
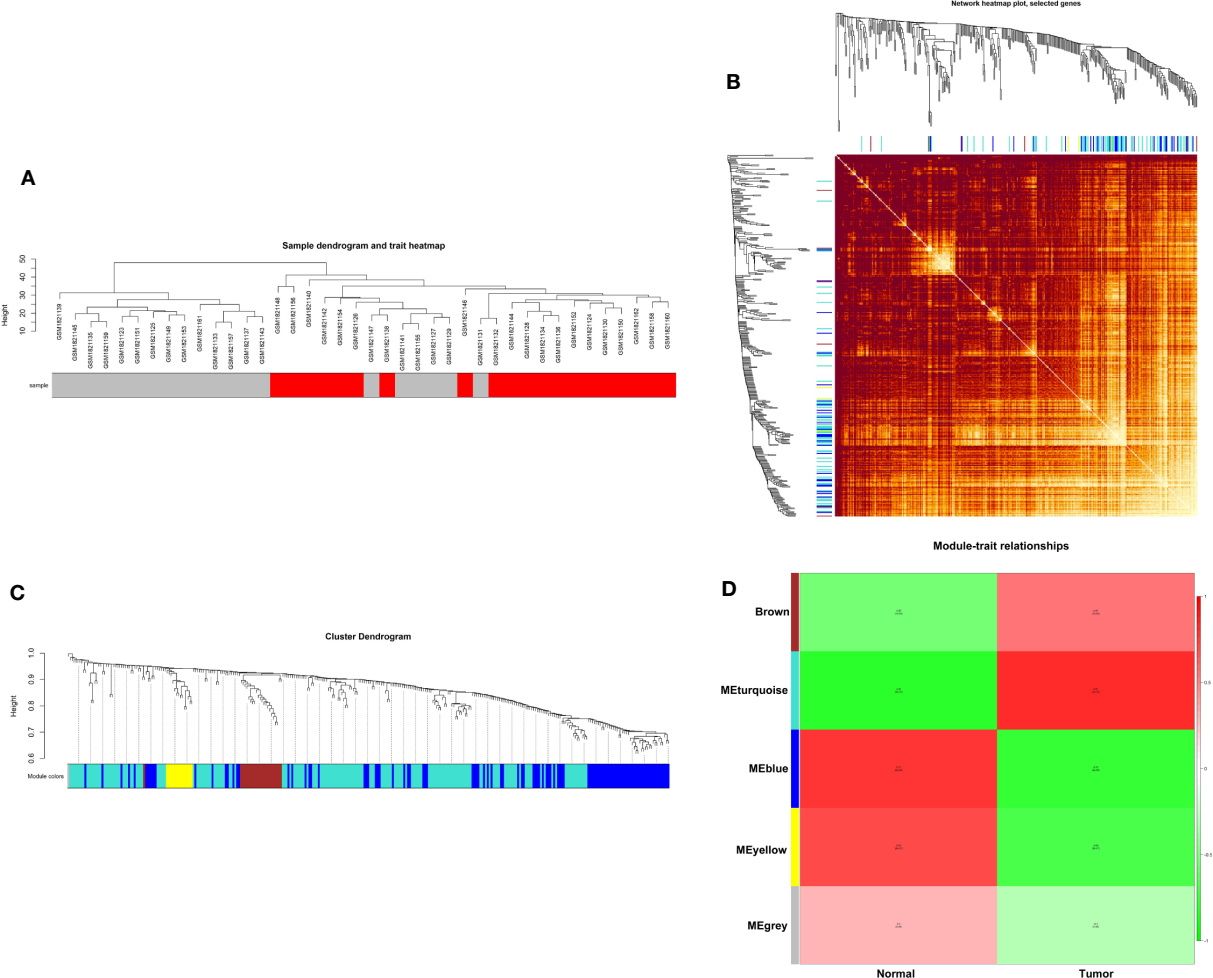
WGCNA of GSE70880. **(A)** Hierarchical clustering dendrogram of the samples. **(B)** Heatmap representing the Topological Overlap Matrix (TOM) among all genes in the WGCNA. **(C)** Average linkage hierarchical clustering dendrogram of the genes. The input was the topological overlap-based dissimilarity. Modules, designated by color code, are the branches of the clustering tree. **(D)** Correlation of module eigengenes to clinical and pathological traits.

**Figure 4:**
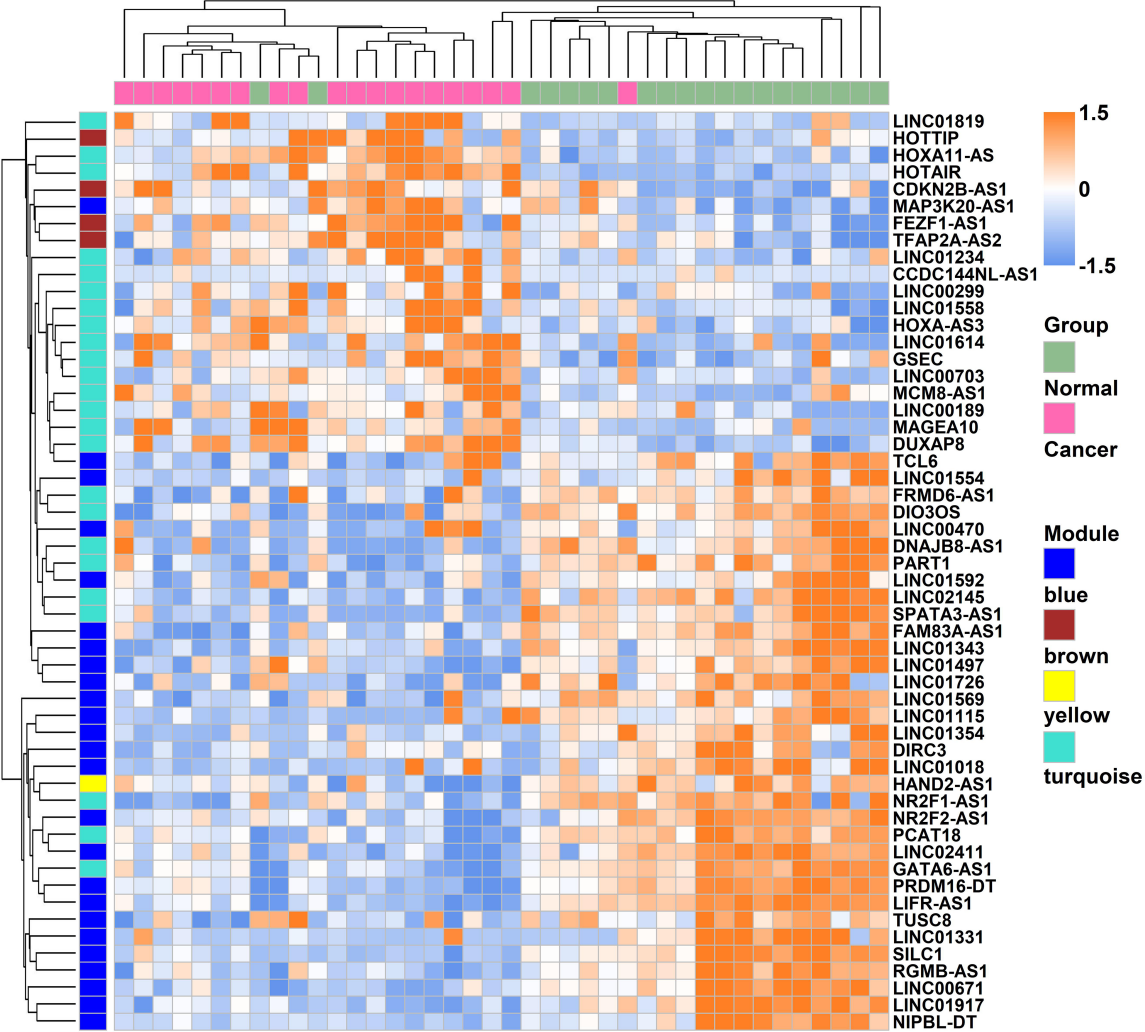
Heatmap of the lncRNAs which were involved in the modules expressed between gastric cancer and normal gastric tissues.

**Figure 5:**
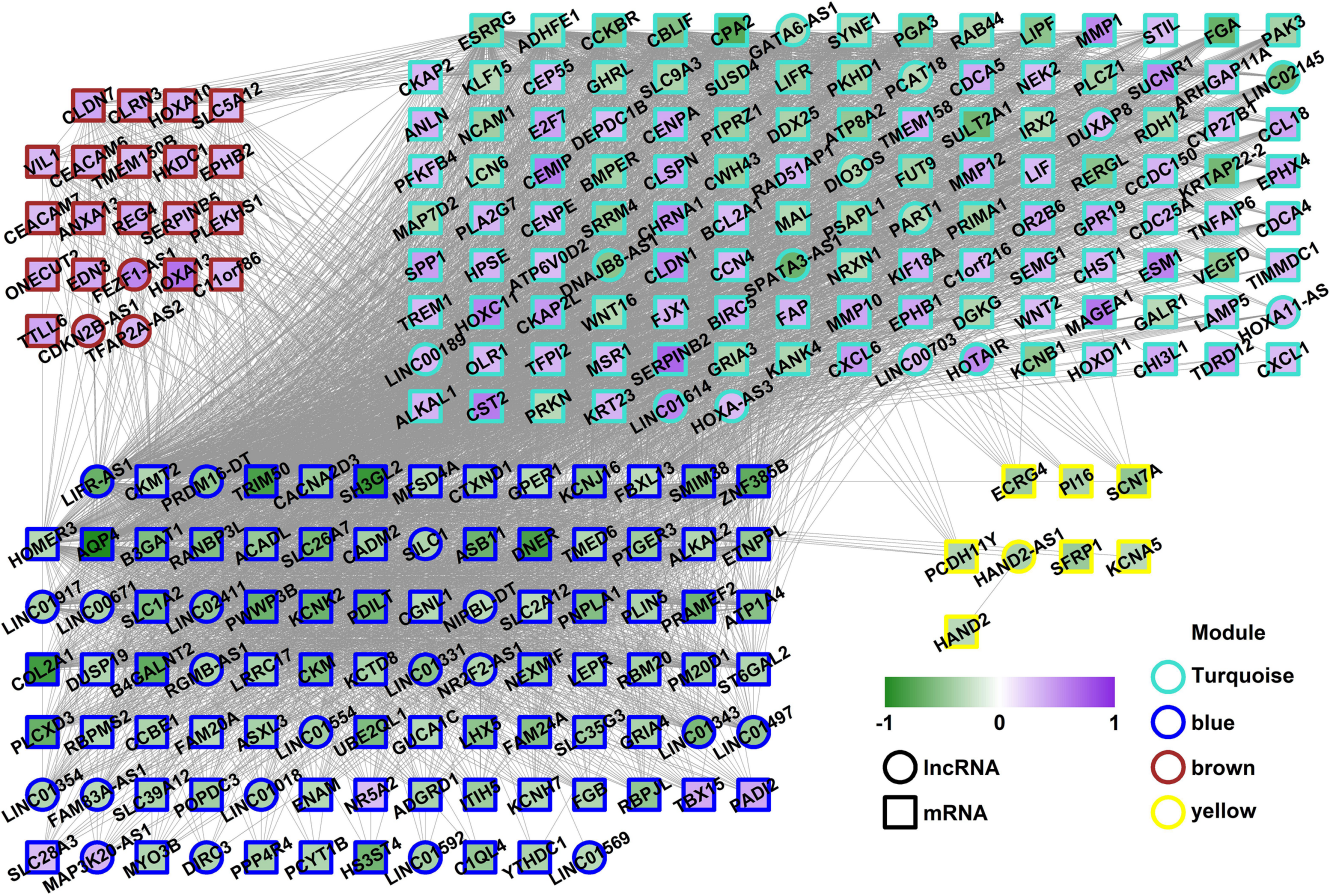
Co-expression network in the different modules. The fill color showed the log2FC of each gene; the border color showed the module the gene was from; the shape showed the type of gene. The square indicates the mRNA and the circle indicates the lncRNA.

Weighted gene co-expression network analysis is a systems biology method to understand correlation patterns among genes across different samples. WGCNA can be used to find clusters or modules. Based on the WGCNA analysis, a total of 5 co-expressed modules are in the analyzed dataset (**Figures 3A–C**). Subsequently, the association between each of the modules and clinical traits (cancer vs normal) was further explored. Based on the analysis, the blue and turquoise modules were strongly correlated with the occurrence of gastric cancer (**Figure 3D**). The differentially expressed lncRNAs in different modules were clustered between the gastric cancer group and the normal gastric tissue group (**Figure 4**). Furthermore, the co-expression network was constructed based on the WGCNA analysis, and the constructed network was demonstrated in **Figure 5**. In addition, **Figure 5** illustrated the hub genes in different modules from the WGCNA analysis.

### Functional Enrichment Analysis of Differentially Expressed Genes in the Different Modules

**Figure 6:**
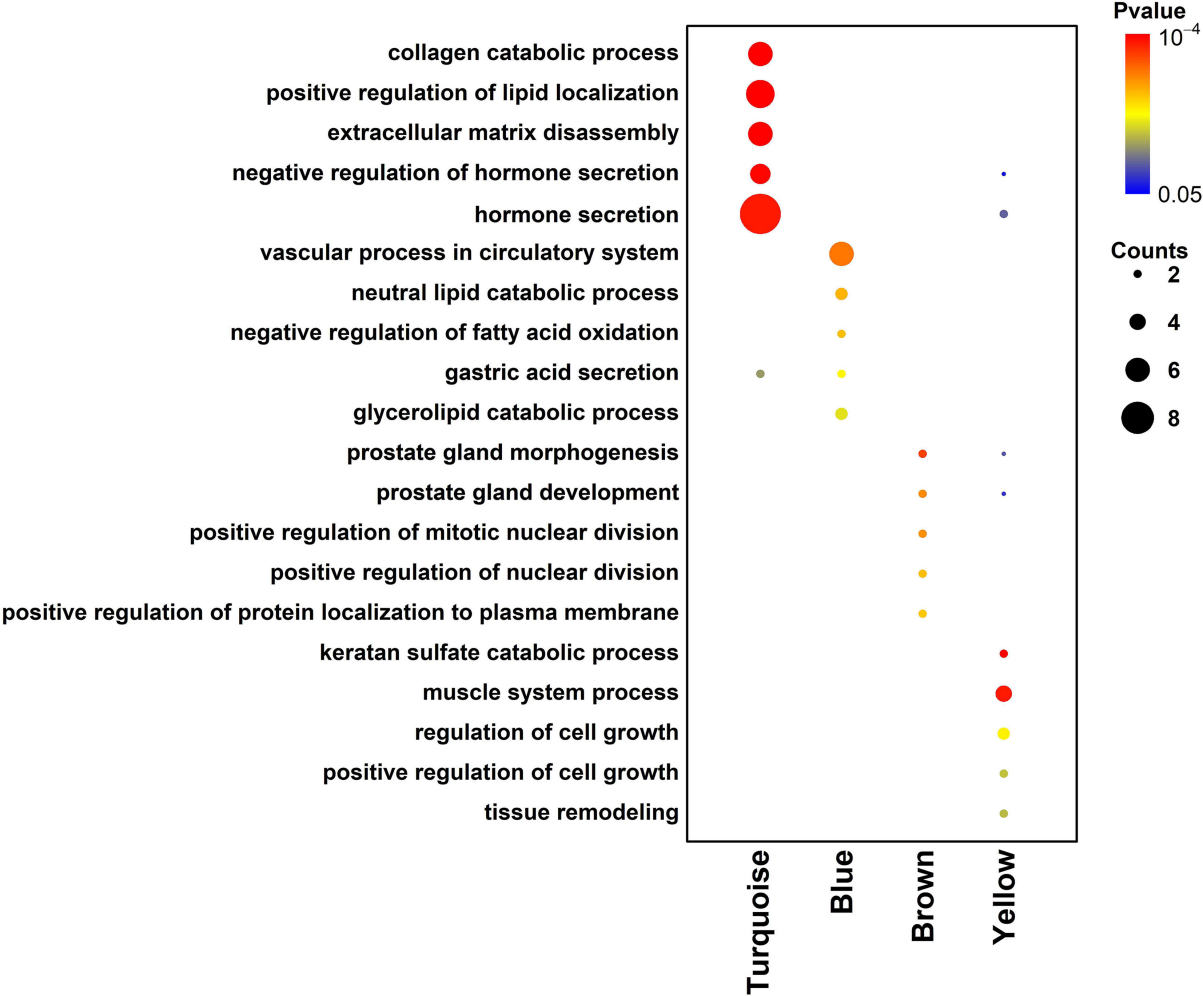
Functional enrichment analysis of differentially expressed genes in the different modules.

The functional enrichment analysis was further performed in the genes from different modules. For the turquoise module, the genes were mainly enriched in “collagen catabolic process”, “positive regulation of lipid localization”, “extracellular matrix disassembly”, “negative regulation of hormone secretion” and “hormone secretion” (**Figure 6**). For the blue module, the genes were mainly enriched in “vascular process in circulatory system”, “neutral lipid catabolic process”, “negative regulation of fatty acid oxidation”, “gastric acid secretion” and “glycerolipid catabolic process” (**Figure 6**). For the brown module, the genes were mainly enriched in “prostate gland morphogenesis”, “prostate gland development”, “positive regulation of mitotic nuclear division”, “positive regulation of nuclear division” and “positive regulation of protein localization to plasma membrane”. For the yellow module, the genes were mainly enriched in “keratan sulfate catabolic process”, “muscle system process”, “regulation of cell growth”, positive regulation of cell growth” and “tissue remodeling”.

### Kaplan−Meier Analysis for the Effects of the lncRNAs on the Survival of Gastric Cancer Patients

**Figure 7:**
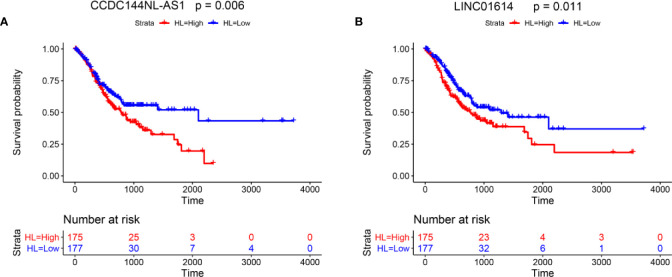
Kaplan−Meier analysis for the effects of the lncRNAs on the survival of gastric cancer patients. Kaplan−Meier survival analysis for **(A)** CCDC144NL-AS1 and **(B)** LINC01614 in gastric cancer patients.

Furthermore, the effects of two lncRNAs (CCDC144NL-AS1 and LINC01614) on the survival of gastric cancer patients were evaluated by Kaplan-Meier analysis. The survival analysis included a total of 352 patients, and high expression of CCDC144NL-AS1 and LINC01614 are both associated with shorter overall survival of patients with gastric cancer (**Figures 7A, B**).

### Effects of CCDC144NL-AS1 and LINC01614 Silence on the Proliferation, Migration and Chemosensitivity of Gastric Cancer Cells

**Figure 8:**
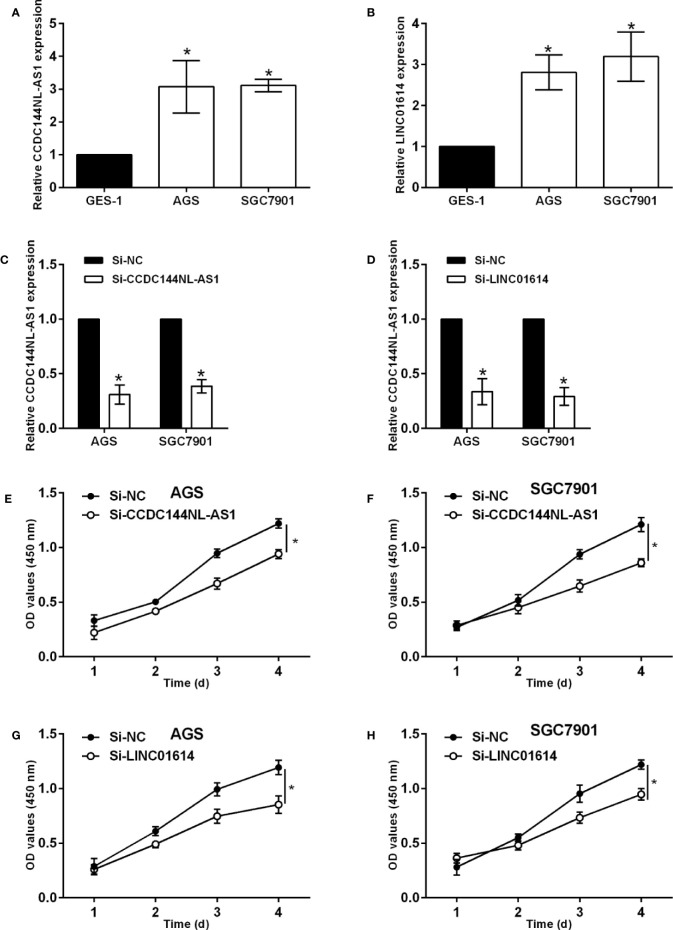
Effects of CCDC144NL-AS1 and LINC01614 silence on gastric cancer cell progression. **(A)** Expression of CCDC144NL-AS1 in GES-1, AGS and SGC7901 cells. **(B)** Expression of LINC01614 in GES-1, AGS and SGC7901 cells. **(C)** Expression of CCDC144NL-AS1 in AGS and SGC7901 cells after siRNAs transfections. **(D)** Expression of LINC01614 in AGS and SGC7901 cells after siRNAs transfections. **(E, F)** Effects of CCDC144NL-AS1 silence on the proliferation of AGS and SGC7901 cells. **(G, H)** Effects of LINC01614 silence on the proliferation of AGS and SGC7901 cells. N = 3. Significant between treatment groups were indicated as *p < 0.05.

**Figure 9:**
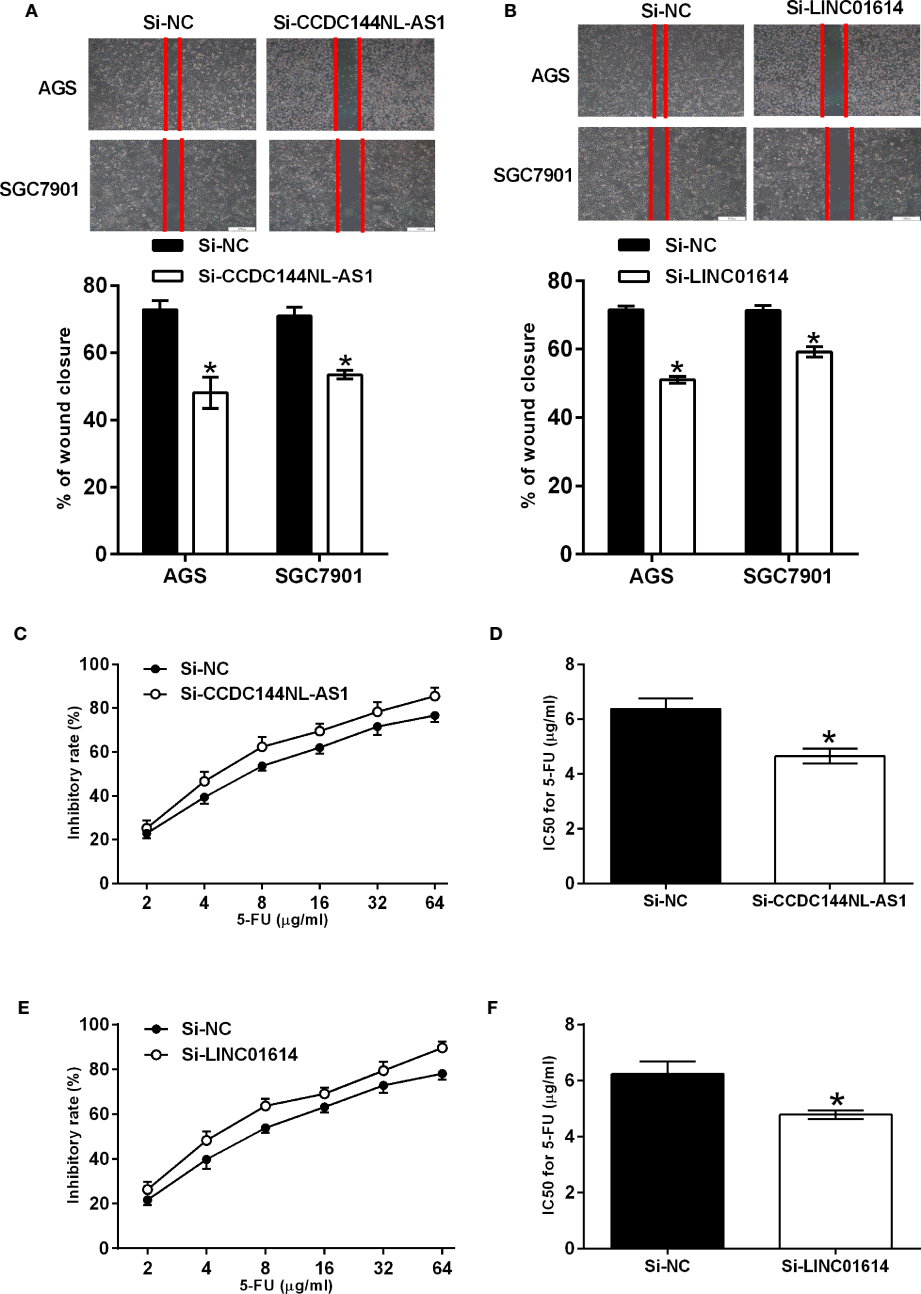
Effects of CCDC144NL-AS1 and LINC01614 silence on the migration and chemosensitivity of gastric cancer cells. **(A)** Effects of CCDC144NL-AS1 silence on the migration of AGS and SGC7901 cells. Top panel shows the representative images of the wound healing area at 24 h after wound scratching. **(B)** Effects of LINC01614 silence on the migration of AGS and SGC7901 cells. Top panel shows the representative images of the wound healing area at 24 h after wound scratching. **(C, D)** Effects of CCDC144NL-AS1 silence on the chemosensitivity of SGC7901/5-FU cells to 5-FU. **(E, F)** Effects of LINC01614 silence on the chemosensitivity of SGC7901/5-FU cells to 5-FU. N = 3. Significant between treatment groups were indicated as *p < 0.05.

The qPCR assay was performed to confirm the expression of CCDC144NL-AS1 and LINC01614 in gastric cancer cells, and CCDC144NL-AS1 and LINC01614 were significantly up-regulated in AGS and SGC7901 cells compared to that in GES-1 cells (**Figures 8A, B**). The knockdown of CCDC144NL-AS1 and LINC01614 in AGS and SGC7901 cells was achieved by transfecting these cells with respective siRNAs for CCDC144NL-AS1 and LINC01614 (**Figures 8C, D**). The CCK-8 assay showed that the silence of CCDC144NL-AS1 and LINC01614 silence both significantly repressed the proliferation of AGS and SGC7901 cells (**Figure 8E–H**). Furthermore, the knockdown of CCDC144NL-AS1 and LINC01614 both impaired the wound closure capacity of AGS and SGC7901 cells (**Figures 9A, B**). The effects of the lncRNAs on the chemosensitivity of gastric cancer cells to 5-FU were determined in SGC7901/5-FU cells. Silence of CCDC144NL-AS1 and LINC01614 both significantly reduced the IC50 values for 5-FU in the SGC7901/5-FU cells (**Figures 9C–F**).

## Discussion

LncRNAs are key regulators in the pathophysiology of gastric cancer, and lncRNAs have been regarded as potential biomarkers and therapeutic targets for gastric cancer (24). With the great advancement in high-throughput technologies, bioinformatic exploration has enabled us to efficiently identify novel lncRNAs associated with cancer progression. In this study, we performed the WGCNA analysis of the GEO dataset (GSE70880), and our results revealed the lncRNA co-expression network. In addition, several hub lncRNAs were detected in the co-expression network based on the WGCNA analysis. The survival analysis showed that high expression of CCDC144NL-AS1 and LINC01614 was positively correlated with the poor prognosis of patients with gastric cancer. The *in vitro* validation results showed that CCDC144NL-AS1 and LINC01614 were both up-regulated in the gastric cancer cells. Silence of CCDC144NL-AS1 and LINC01614 both significantly suppressed the cell proliferation and migration of gastric cancer cells, and also promoted the chemosensitivity of gastric cancer cells to cisplatin. Collectively, our results suggested that the newly identified two lncRNAs (CCDC144NL-AS1 and LINC01614) may act as oncogenes in gastric cancer.

WGCNA is a system biology strategy to reveal correlation patterns among genes across different samples. This analysis is useful in identifying the modules or clusters. In addition, the eigengene can be used to summarize modules, and examine the correlation between the modules and sample traits. Le et al., performed the WGCNA using the GSE76250 dataset and revealed a novel competing endogenous RNA network for triple-negative breast cancer (25). Qian et al., performed the analysis of lncRNA-mRNA networks after MEK1/2 inhibition based on WGCNA in pancreatic cancer and found that NONHSAT185150.1 and beta-1,4-galactosyltransferase were negatively correlated with MEK1/2 (26). Consistently, Giulietti et al., performed a similar analysis and revealed novel biomarkers (LINC00675 and LINC01133) for pancreatic cancer (21). Based on the WGCNA analysis, Li et al., demonstrated that lncRNA51663 and FLJ46906 were remarkably increased in H. pylori-infected cells and consistently overexpressed in human gastric cancer tissues compared to adjacent normal tissues (27). In agreement with previous studies, we performed the WGCNA on the GSE70880 dataset and identified two novel lncRNAs (CCDC144NL-AS1 and LINC01614) that may be associated with the survival of patients with gastric cancer.

The role of CCDC144NL-AS1 has been implicated in several types of cancers. Zhang et al., showed that knockdown of CCDC144NL-AS1 attenuated migration and invasion phenotypes in endometrial stromal cells from endometriosis (28). CCDC144NL-AS1 also promoted the oncogenicity of osteosarcoma by acting as a molecular sponge for microRNA-490-3p and thereby increasing HMGA2 Expression (29). In addition, Zhang found that CCDC144NL-AS1 could promote the development of hepatocellular carcinoma by inducing WD repeat domain 5 expression *via* sponging miR-940 (30). A recent study demonstrated that CDC144NL-AS1 served as a prognosis biomarker for non-small cell lung cancer and promoted cellular function by targeting miR-490-3p (31). However, the role of CDC144NL-AS1 in gastric cancer progression remains unknown. Our *in vitro* studies showed that silence of CDC144NL-AS1 inhibited the progression of gastric cancer cells and enhanced the chemosensitivity of gastric cancer cells to cisplatin, suggesting that CDC144NL-AS1 may serve as an oncogenic RNA in gastric cancer.

The functional role of LINC01614 in cancer progression was first reported by Liu et al., and the study found that LINC01614 suppressed lung cancer cell progression by regulating miR-217 and down-regulating forkhead box P1 (32). Vishnubalaji et al., performed the lncRNA transcriptional analysis in breast cancer and identifies LINC01614 as a non-favorable prognostic biomarker regulated by transforming growth factor beta and focal adhesion kinase signaling (33). Consistently, Wang et al., found that LINC01614 could serve as a potential biomarker for prognostic prediction in breast cancer (34). In glioma, Wang et al., found that SP1-mediated upregulation of LINC01614 functions a ceRNA for miR-383 to facilitate tumor progression *via* regulation of ADAM12 (35). Recently, Cai et al., showed that LINC01614 promoted osteosarcoma progression *via* the miR-520a-3p/sorting nexin 3 axis (36). Our *in vitro* studies showed that silence of LINC01614 attenuated the progression of gastric cancer cells and enhanced the chemosensitivity of gastric cancer cells to cisplatin, suggesting that LINC01614 may serve as an oncogenic RNA in gastric cancer.

This study has several limitations for consideration. Firstly, the WGCNA was performed in only one dataset, and future studies may consider analyzing more datasets to reveal more novel lncRNAs associated with gastric cancer progression. Secondly, the prognostic role of the newly identified lncRNAs has not been examined in the clinical studies, which should be explored in future studies. Thirdly, the investigation into the mechanistic role of CCDC144NL-AS1 and LINC01614 is still at the early stage, and more mechanistic studies should be considered, to reveal the potential actions of these two lncRNAs in gastric cancer.

## Conclusions

In conclusion, our study performed the WGCNA and revealed that CCDC144NL-AS1 and LINC01614 might be potential biomarkers for the prognosis of gastric cancer patients. Further *in vitro* functional studies indicated that CCDC144NL-AS1 and LINC01614 might serve as oncogenic lncRNAs in gastric cancer. The present study for the first time provides novel insights into the role of CCDC144NL-AS1 and LINC01614 in the pathophysiology of gastric cancer.

## Data Availability Statement

The original contributions presented in the study are included in the article/supplementary material. Further inquiries can be directed to the corresponding author.

## Author Contributions

YZ designed and supervised the whole project. WS and WZ performed the experiments and summarized the data. YZ wrote the manuscript. YZ revised the drafted manuscript. All authors contributed to the article and approved the submitted version.

## Funding

The work was supported by grants from the Scientific Research Project of Jiangsu Provincial Health Committee (Z2021056).

## Conflict of Interest

The authors declare that the research was conducted in the absence of any commercial or financial relationships that could be construed as a potential conflict of interest.

## Publisher’s Note

All claims expressed in this article are solely those of the authors and do not necessarily represent those of their affiliated organizations, or those of the publisher, the editors and the reviewers. Any product that may be evaluated in this article, or claim that may be made by its manufacturer, is not guaranteed or endorsed by the publisher.
